# Gas-powered extracellular vesicles promote bone regeneration

**DOI:** 10.20517/evcna.2024.91

**Published:** 2025-03-19

**Authors:** Lexie Shannon Holliday, John K. Neubert, Xianrui Yang

**Affiliations:** Department of Orthodontics, University of Florida College of Dentistry, Gainesville, FL 32610, USA.

**Keywords:** Osteogenesis, exosomes, hydrogen sulfide, moesin, Wnt/β-catenin, macrophage, osteoblast

## Abstract

The signaling gas hydrogen sulfide (H_2_S) has recently been implicated in the regulation of bone remodeling and the maintenance of periodontal health. Exploring the underlying mechanisms for this regulation holds promise for the development of new treatment strategies to block bone resorption and stimulate bone regeneration. A recent study by Zhou *et al.* (Bioactive Materials, 2024) showed that treatment with H_2_S stimulated changes in the extracellular vesicles (EVs) released by M2 macrophages, enhancing their capacity to promote the osteogenic differentiation of mesenchymal stem cells *in vitro.* The H_2_S-stimulated EVs, given together with mesenchymal stem cells (MSCs), also promoted bone regeneration *in vivo* in a mouse calvarial critical-size defect model. This activity was linked to augmented expression of moesin, a membrane-cytoskeletal linkage protein, which was found at increased levels in EVs from cells stimulated by H_2_S. The study identifies a new strategy for generating EVs that are pro-osteogenic. It also uncovers a surprising role for moesin in stimulating osteogenesis in MSCs.

## COMMENTARY

Until 1996, hydrogen sulfide (H_2_S) was considered a toxic gas. However, that perception changed when Abe and Kimura showed that H_2_S, along with its producing enzyme cystathionine β-synthase, is highly expressed in the hippocampus. They found that H_2_S produced by cystathionine β-synthase selectively enhances N-methyl-D-aspartate (NMDA) receptor-mediated responses and facilitates the induction of hippocampal long-term potentiation^[1]^. Subsequent studies identified cystathionine γ-lyase and 3-mercaptopyruvate sulfurtransferase as additional H_2_S-generating enzymes in mammalian cells, thereby completing the enzymatic system responsible for H_2_S production^[2]^. This discovery positioned H_2_S alongside nitric oxide (NO) and carbon monoxide (CO) as a signaling gas^[3]^. H_2_S has since been shown to mediate vasodilation^[4]^, exhibit anti-tumor and anti-metastatic properties, and regulate bone remodeling^[3,5,6]^. In addition to its role in enhancing NMDA receptor-mediated responses, several mechanisms have been suggested to account for the signaling effects of H_2_S, including the activation of ATP-sensitive potassium channels^[7,8]^, the stimulation of endothelial nitric oxide synthase (eNOS) to increase NO production^[9]^, and the inhibition of voltage-gated calcium channels^[10]^. By inducing S-sulfhydration of a ubiquitin-ligase, H_2_S was shown to regulate CD36 vesicle transport in diabetic hearts^[11]^. H_2_S has been reported to have either pro-inflammatory or anti-inflammatory effects^[12,13]^. It has been implicated in regulating macrophages during inflammatory stimulation^[14]^. Additionally, it was recently shown to be involved in anti-viral responses, including the response to SARS-CoV-2^[15]^. Because of its potential therapeutic activities, small molecule H_2_S donors have been developed and are being tested as therapeutic tools^[6]^.

**Figure 1:**
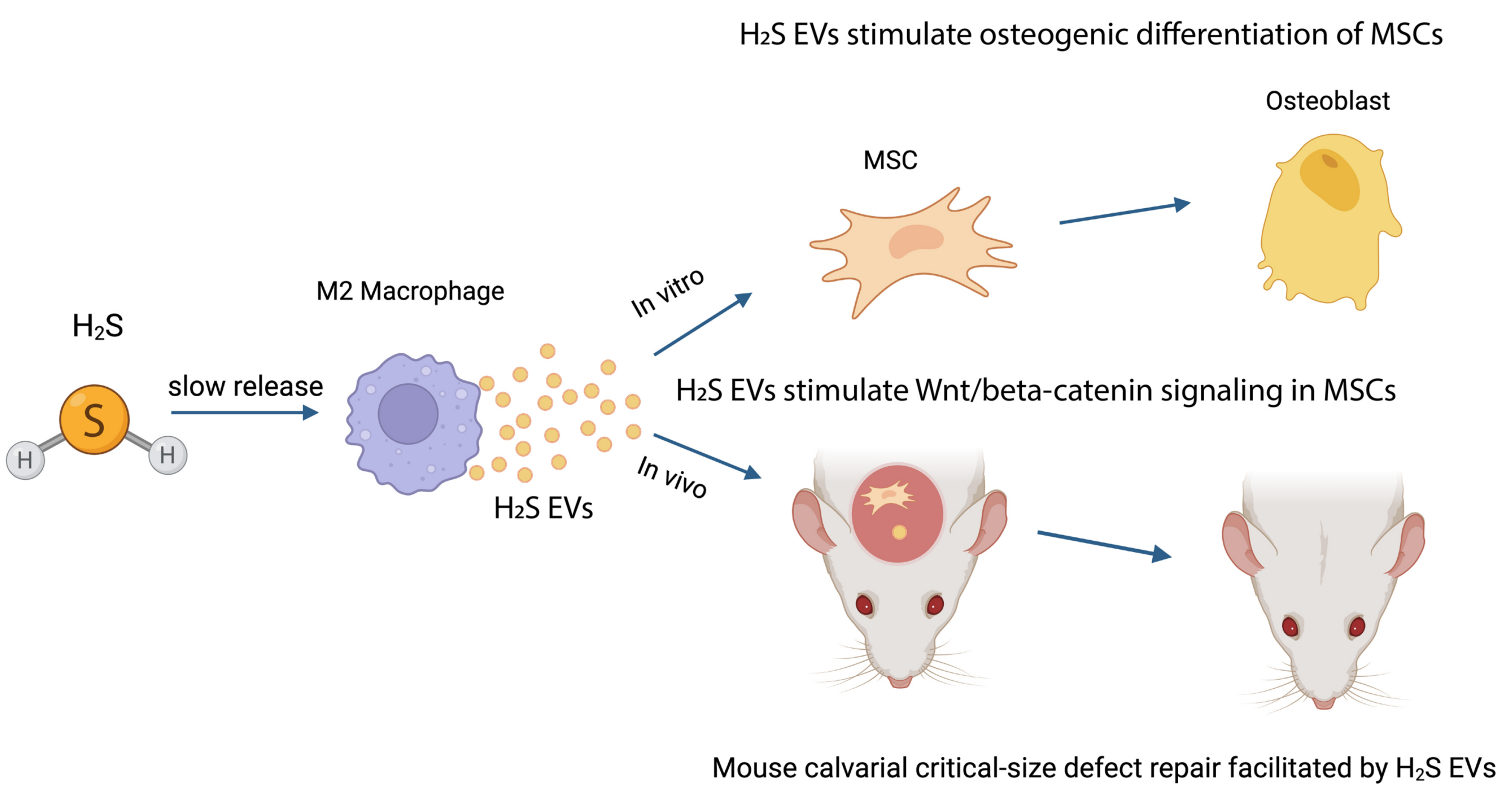
Schematic of findings of Zhou *et al.*^[16]^. H_2_S-stimulated M2 macrophages produce EVs that have increased ability to promote differentiation of MSCs into osteoblasts *in vitro*. When H_2_S-stimulated EVs were incorporated into Matrigel along with MSCs, which was then used to fill calvarial critical-size defects in mice, bone regeneration occurred significantly more quickly than when unstimulated M2 macrophage EVs or no EVs were used with MSCs. H_2_S: Hydrogen sulfide; EVs: extracellular vesicles; MSCs: mesenchymal stem cells.

This commentary is focused on a recent study by Zhou *et al.* that described a novel mechanism by which H_2_S regulates bone regeneration^[16]^ [Figure 1]. It was reported that a slow-release donor of H_2_S, GYY4137, stimulated the polarization of M2 macrophages and changes in the composition of extracellular vesicles (EVs) released from these cells. In the manuscript, the authors referred to the extracellular vesicles as exosomes. However, based on the data they presented and by the International Society of EVs recommendations^[17]^, I will refer to these vesicles as EVs throughout this commentary. H_2_S-stimulated EVs from M2 macrophages, together with mesenchymal stem cells (MSCs), increased bone formation when used in a scaffold to fill critical size defects in mouse calvaria. Liquid Chromatography/Mass spectroscopy was employed to examine the protein composition of EVs from M2 macrophages with or without H_2_S treatment. From this analysis, the membrane-cytoskeleton linkage protein moesin was identified as a candidate for mediating the increased osteogenic effects. RNA interference knockdown of moesin in M2 macrophage resulted in EVs that were less able to exert osteogenic effects on MSCs. Surprisingly, recombinant moesin added to MSCs increased osteogenic differentiation. Taken together, these data suggest that pre-treating M2 macrophages with H_2_S induces them to secrete EVs that have therapeutic potential for use in situations where bone regeneration is required. Moesin is a component of the mechanism of action leading to this regulatory activity.

Previous studies have shown that M2 macrophages produce paracrine factors that promote osteogenesis^[18-20]^. Of these paracrine factors, several groups reported that EVs were key contributors^[21-26]^. In the current study, it was reported that H_2_S conditioned media from M2 macrophages stimulated osteogenic differentiation of MSCs. Evidence included increased expression of alkaline phosphatase and runt-related transcription factor 2 (*RUNX2*) genes, increased alkaline phosphatase enzymatic activity, and more deposition of mineral crystals *in vitro*. These experiments confirmed previous reports and provided a solid experimental platform for further experimentation.

Having found that conditioned media from M2 macrophages promotes osteogenesis, Zhou *et al.* then tested whether pre-treatment of the macrophages with H_2_S would affect the regulatory activity of the conditioned media^[16]^. Their data showed the conditioned media from H_2_S-stimulated M2 macrophages displayed a significantly increased ability to promote osteogenic differentiation of MSCs.

EVs were then isolated from the conditioned media by differential centrifugation. It was confirmed that the protocol employed yielded EVs using standard techniques including electron microscopy, nanoparticle tracking, and immunoblots for markers of EVs and of potential contaminants. They found that H_2_S stimulation did not significantly change the number of EVs produced. They characterized EVs, comparing EVs from macrophages, M2 macrophages, or H_2_S-stimulated M2 macrophages. Among their findings were that EVs for H_2_S-stimulated M2 macrophages showed an increased ability to be taken up by target MSCs. This was measured by labeling the EVs with PKH26, a fluorescent membrane vital dye, and then incubating the labeled EVs with MSCs. Although this does not provide quantitative information regarding the absolute efficiency of EV incorporation by the MSCs, it does suggest that EVs from H_2_S-stimulated M2 macrophages are taken up more efficiently relative to the EVs from unstimulated cells.

EVs from both M2 macrophages and H_2_S-stimulated M2 macrophages promoted osteogenesis in the MSCs *in vitro*, with effects comparable to those induced by conditioned media. The EVs were then tested *in vivo* by mixing them with MSCs in Matrigel, which was used to fill critical-sized defects in mice. The H_2_S-stimulated M2 macrophage EVs in Matrigel with MSCs stimulated bone regeneration in the defects better than the same amount of unstimulated M2 macrophage EVs, or Matrigel alone.

To examine the underlying mechanism, differences in the proteins found in the M2 macrophage-derived EVs were compared with the EVs from the macrophages treated with H_2_S. This analysis suggested the involvement of the membrane-cytoskeletal linkage protein, moesin, which was found at higher levels in EVs from the H_2_S-stimulated M2 macrophage. To test the role of moesin, RNA interference was used to knock down the expression of moesin in the H_2_S-treated M2 macrophages. EVs from moesin knockdown cells displayed decreased osteogenic stimulation. Evidence was provided that the EVs were acting by stimulating wingless-related integration site (Wnt)/β-catenin signaling, which promoted osteogenesis. Interestingly, the simple addition of recombinant moesin to the MSCs also increased osteogenesis.

Moesin is a membrane cytoskeletal linkage protein that is often found in filopodia^[27]^. It is a member of the ezrin-radixin-moesin (ERM) family of linkers^[28]^. Moesin is thought to be located on the cytoplasmic face of the plasma membrane; in EVs, it would be expected to be in the lumen, and thus not exposed to the surface of the target cell. There are also reports of moesin entering the nucleus, where it is involved in gene expression and mRNA export^[29]^.

Zhou *et al.* hypothesized that moesin in EVs promoted the uptake of EVs by MSCs, which was required for the promotion of osteogenic differentiation^[16]^. In our view, moesin could act at various levels to promote EV uptake based on its known activities. First, moesin is involved in the regulation of focal adhesions^[30]^. Changes in this regulation could alter regulatory EVs. We found that EVs from osteoclasts on different substrates have different protein compositions and regulatory activity due to the signals received from the extracellular matrix^[31]^. This could be consistent with moesin acting at this level. Indeed, we detected moesin at high levels in EVs from osteoclasts on bone or odontoclasts on dentine, but at much lower levels in EVs from osteoclasts on plastic^[31]^. Second, moesin could be involved in EV formation. Unfortunately, the number of EVs isolated from moesin knockdown cells compared with moesin-replete cells was not reported. Third, moesin could be directly involved in sorting regulatory proteins or other molecules into EVs. A proteomic study of moesin-knockdown EVs would be helpful in testing this idea. Fourth, moesin could facilitate the targeting and uptake of EVs to MSCs. For example, moesin could be a component of a stabilizing complex for a receptor that enables EV uptake. Evidence for EVs containing stabilizing complexes for receptors was discussed in detail in a recent review^[32]^. Fifth, once incorporated into MSCs, moesin could facilitate the formation of a signaling complex in the MSCs. Along that line, it is intriguing that the prorenin receptor (ATP6AP2), which is found at high levels in EVs from osteoclasts - cells related to macrophages - was implicated as a component of the Wnt/β-catenin signaling complex^[33]^. Moesin activates Wnt/β-catenin by regulating the phosphorylation state of CD44 and the trafficking of CD44 to the plasma membrane^[34,35]^. Perhaps when EVs from M2 macrophages fuse with MSCs, they supply multiple components (moesin, prorenin receptor) that stimulate Wnt/β-catenin signaling. Sixth, moesin introduced into the cytosol of target cells after EV fusion could be transported to the nucleus, where it could alter gene expression or mRNA export^[29]^.

As described above, in the report, recombinant moesin added to MSCs increased osteogenic differentiation. This experiment is difficult to reconcile with the hypothesized mechanism and known functions and location of moesin in cells as described above. This finding may point to a new activity of moesin. This conclusion requires significant further verification, but other cytoskeletal proteins, including thymosin beta-4, have roles both inside and outside the cell^[36]^.

Before Zhou *et al*.^[16]^, a recent study had shown that treatment of MSCs with H_2_S resulted in variations in the composition of the EVs the cells produced and changes in the EVs’ regulatory activity^[37]^. Currently, a concerted effort by both the pharmaceutical industry and basic scientists is underway to explore ways to enhance or inhibit the production of regulatory EVs. Various agents have also been reported to alter EV composition and regulatory activity. Our group, as described above, reported that the substrate osteoclasts adhere to regulates the overall protein composition of EVs produced by 10%-30%, and importantly, the abundance of the regulatory molecule RANK^[31,38]^. A high-throughput screen of 4,580 pharmacologically active compounds identified 22 that were potent activators or inhibitors of EV production^[39]^. Although this seems a small portion, the assay used in this study only detected changes in CD63-containing EVs, which was accomplished by linking CD63 to green fluorescent protein and then screening for fluorescence in EV fractions. The same sort of assay could be used for cells producing specific regulatory molecules (for example, moesin) found in EVs. Based on Zhou *et al.*’s study, H_2_S would not be found to generally increase EV production and would not be detected in the high throughput assay for CD63-containing EVs described above, but instead would alter the regulatory molecules present, including moesin, and would be detected by a screen for moesin levels^[16]^.

In addition to H_2_S, NO has been shown to regulate the activity of EVs, increasing the pro-angiogenic effects of EVs from MSCs^[40]^. Like H_2_S and H_2_S donors, NO and NO donors have the capacity to modulate regulatory EV production in at least some cell types. Understanding how specific signaling molecules and therapeutic agents affect regulatory EVs is at a very early stage, and Zhou *et al*. represent a significant contribution to this ongoing effort^[16]^.

## STRENGTHS, LIMITATIONS, AND FUTURE DIRECTIONS

A primary strength of the study was the multi-level approach, examining the effects of H_2_S *in vitro* and *in vivo*. The *in vitro* approach included proteomic assessment, which identified a potential mechanistic element in moesin. In most cases, the experiments were well done, using multiple confirmatory approaches to increase confidence in the result. An important weakness was the quantitation of EVs by protein concentration. During EV isolations, contaminating proteins like albumin from serum are often the major protein component in EV preparations. This makes replication of the experiments described a problem. The finding that recombinant moesin added to MSCs increased osteogenesis is surprising and potentially of great importance because it is very unexpected. Follow-up experiments, such as determining the domain of moesin responsible for the activity, would be exciting. Indeed, showing that soluble moesin is an important intercellular regulatory molecule could be the most generally important finding from the study. Studies examining changes in the number and composition of EVs after moesin knockdown would also be useful. Finally, Zhou *et al.* did not address the signaling pathway through which H_2_S triggered changes in EVs^[16]^. NMDA agonists and antagonists are available and could be used to test whether H_2_S affects EV production by NMDA receptors.

Future work that follows Zhou *et al*.’s includes testing whether H_2_S has a role in the physiological regeneration of bone^[16]^. Studies showing a physiologic role of H_2_S in orthodontic tooth movement, orthodontic root resorption, and periodontal disease suggest that this may be the case^[41-43]^. Even if H_2_S is not normally involved in bone regeneration under physiological conditions, the current data indicate that stimulation of pro-osteogenic EVs from M2 macrophages may be a useful approach to enhancing bone regeneration. A crucial experiment to understand the underlying mechanism of the EVs is to determine the absolute efficiency of EV fusion with target cells, and whether luminal components of EVs enter the cytosol of the MSCs. The signaling mechanism by which H_2_S affects EV production is also vitally important. For example, if H_2_S acts through NMDA receptors, then existing NMDA agonists could be used to stimulate the osteogenic EVs^[44]^. It will be crucial to work out the mechanism by which these EVs and moesin are involved in stimulating the osteogenic differentiation of MSCs in detail. Such work could lead to “next-generation” therapeutic strategies for bone regeneration and other clinical challenges.

## DECLARATIONS

### Copyright

© The Author(s) 2025.
